# Associations between changes in the pattern of suicide methods and rates in Korea, the US, and Finland

**DOI:** 10.1186/1752-4458-8-22

**Published:** 2014-06-04

**Authors:** Subin Park, Myung Hee Ahn, Ahrong Lee, Jin Pyo Hong

**Affiliations:** 1 Department of Psychiatry, Seoul National Hospital, 398 Neungdong-ro, Gwangin-gu, Seoul, South Korea; 2 Department of Psychiatry, Asan Medical Center, Ulsan University College of Medicine, 388-1 Pungnap-2dong, Songpa-gu, Seoul 138-736, South Korea

**Keywords:** Suicide, Method, Hanging

## Abstract

**Background:**

The lethality of the suicide method employed is a strong risk factor for the completion of suicide. We examined whether annual changes in the pattern of suicide methods is related to annual changes in suicide rates in South Korea, the United States (US), and Finland.

**Methods:**

We analyzed annual data from 2000–2011 for South Korea and Finland, and 2000–2010 for the US in order to examine trends in the rates and methods of suicide. Data on suicide methods were obtained from the World Health Organization (WHO) mortality database.

**Results:**

Along with an annual rapid increase in suicide rates, the incidence of hanging increased steadily while suicide by self-poisoning steadily decreased in South Korea. In the US, along with an annual increase in suicide rates, the proportion of suicides committed by hanging increased while those committed with the use of firearms steadily decreased. In Finland, annual changes in the suicide rate and suicide method were not statistically significant during the study period.

**Conclusions:**

Our present findings suggest that the increased use of specific lethal methods for suicide, namely hanging, is reflected in the increased suicide rates in the Korean and the US populations. The most effective approach for reducing overall suicide rates may be the implementation of population-based initiatives that reduce both the accessibility (e.g., access to firearms) and the social acceptability (e.g., effective and responsible regulations for reporting suicide) of lethal methods of suicide.

## Background

In 2012, South Korea had the highest suicide rate of all countries in the Organization for Economic Cooperation and Development (OECD) [1]. In recent years, suicide rates have increased rapidly and steadily in South Korea: in 1990 an average of 9.8 suicides per 100,000 individuals were observed, and in 2012 the rate was 33.5 suicides per 100,000 individuals.

The lethality of the chosen suicide method is a strong risk factor for suicide completion [2]. Use of firearms and hanging are two of the most lethal suicide with over 80% case fatality while self-poisoning and self-injurious behavior with sharp objects having lower fatality (less than 5%) [2,3]. National studies on suicide indicate that the preferred suicide method varies between countries. Some patterns are well known, such as the high proportion of firearm suicides in the United States [4] and the prevalent pesticide suicide in Asian countries in the 1990s [5]. Hanging was the most prevalent suicide methods in many countries including European countries [6], Australia [7], and Canada [8]. In South Korea, use of firearms is very rare, because of strict government restrictions limiting firearms [9]. Hence, a trend toward increased use of hanging among Koreans may partially explain the increased suicide rates.

In our present study, we examined whether annual changes in the pattern of suicide methods is related to the annual change in suicide rates in South Korea. We used suicide victims in the United States and Finland as the comparison group for an investigation of trends in suicide rates and methods in South Korea. The United States, where guns are more available, is selected to examine the effect of use of firearms, one of the most lethal method, on suicide rates. Finland is selected because it is the first country to establish a research-based comprehensive national program for suicide prevention, which significantly decreased national suicide rates and earned praises from around the world [10]. In the United States, the suicide rate has increased steadily since 2000 (10.4 suicides per 100,000 individuals), with 12.6 suicides per 100,000 individuals in 2010. However, the extent of change in suicide rates was not as rapid in the United States compared with South Korea. In Finland, the suicide rate has decreased since 1990, with 30.3 suicides per 100,000 individuals in 1990, 22.5 suicides per 100,000 individuals in 2000, and 16.9 suicides per 100,000 individuals in 2011 [11]. We hypothesize that the increased use of more lethal methods such as hanging and firearms may be associated with the increase in the total suicide rates, and that this association may be more prominent in countries where the use of less lethal method is replaced by more lethal method, than in countries where the use of one specific lethal method of suicide is replaced by other specific lethal method.

## Methods

We analyzed annual data during the period 2000–2011 to examine trends in suicide rates and suicide methods in three countries. Data on suicide rates and methods among people aged 10 years over were obtained from the World Health Organization (WHO) mortality database [11]. The most recent data were from 2011 for South Korea and Finland, and from 2010 for the United States. Suicide methods were classified into five categories according to the ICD-10 codes: self-poisoning (X60–X69), hanging (X70), firearms (X72–74), jumping from a high place (X80), and others (X71, X75–X79, X81–X84).

The WHO obtains data on deaths that includes age, sex, and cause of death, as reported annually by member states through civil registration systems (e.g., the Korean National Statistical Office, the National Center for Health Statistics of the United States, and the Statistics Finland), and compiles the data in the WHO mortality database. Based on country-reported data and the use of additional data sources such as population censuses and household surveys, the WHO and its partners regularly estimate key mortality statistics to improve data comparability across countries and years. In addition, the WHO estimates the completeness and coverage of reported data, and assesses the quality of cause-of-death data. According to the WHO, the rate of civil registration coverage of cause of death was 100% in all three countries. The rate of ill-defined causes in cause-of-death registration was 15.4% in South Korea, 6.7% in the United States, and 2.4% in Finland in 2010.

We performed Spearman correlation analyses to assess trends in suicide rates over the 12-year period and potential correlations between specific suicide methods and suicide rates. All statistical analyses were performed using SPSS (version 21.0; SPSS Inc., Chicago, IL), with statistical significance defined as an alpha level of 0.05/(5 types of suicide methods × 3 countries) = 0.0033.

## Results

**Figure 1:**
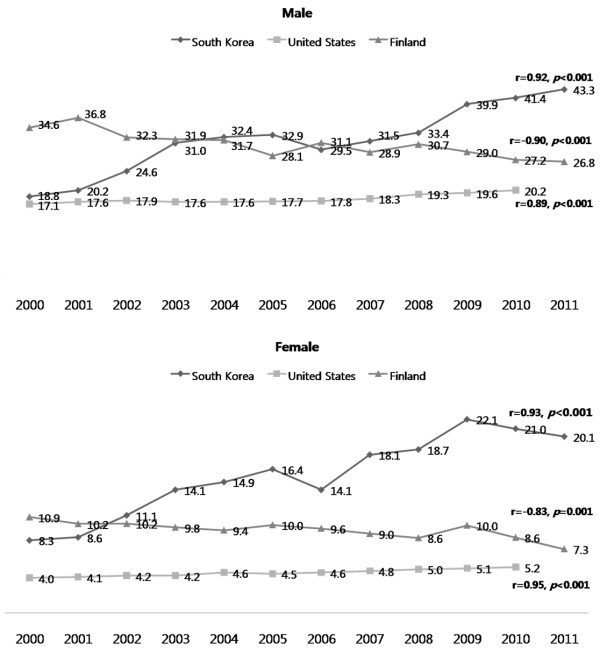
Annual trends in suicide rates in South Korea, the United States, and Finland.

Annual suicide rates in South Korea and Finland between 2000 and 2011 as well as annual suicide rates in the United States between 2000 and 2010, are shown in Figure 1. During this period, suicide rates for males in South Korea rapidly increased from 18.8 to 43.3 suicides per 100,000 individuals, and from 8.3 to 20.1 suicides per 100,000 individuals for females. On the other hand, suicide rates among the Finnish population decreased from 34.56 to 26.76 suicides per 100,000 individuals for males and 10.94 to 7.29 suicides per 100,000 individuals for females. The United States suicide rate slightly but steadily increased from 17.1 to 20.2 suicides per 100,000 individuals for males and from 4.0 to 5.2 suicides per 100,000 individuals for females.

**Figure 2:**
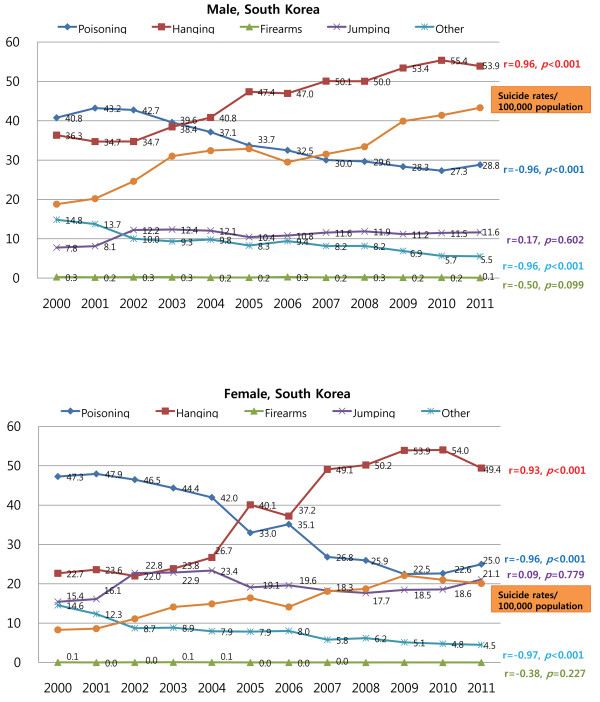
Trends in the suicide methods employed by Korean males and females between 2000 and 2011.

In South Korea, self-poisoning was the most common method of suicide for both males and females until the early 2000’s. The rate of self-poisoning significantly and steadily decreased since 2001 (r = −0.96, *P* < 0.001 for both males and females). In contrast, the rate of hanging significantly increased (r = −0.96, *P* < 0.001 for males and r = 0.93, *P* < 0.001 for females), and hanging has been the most common method of suicide among Korean males since 2003 and among Korean females since 2005 (Figure 2). For both Korean males and females, there was a significant positive correlation between suicide rate and the rate of hanging (r = 0.90, *P* < 0.001 and r = 0.95, *P* < 0.001, respectively), and a negative correlation between suicide rate and both the rate of poisoning (r = −0.88, *P* < 0.001 and r = −0.98, *P* < 0.001, respectively) and the rate of other methods (r = −0.92, *P* < 0.001 and r = −0.95, *P* < 0.001, respectively).

**Figure 3:**
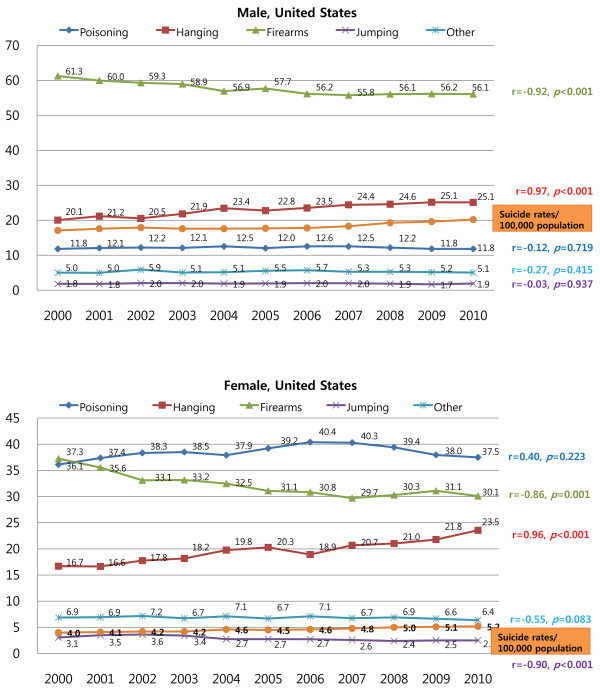
Trends in the suicide methods employed by American males and females between 2000 and 2010.

During the study period, firearms were the most common method of suicide in American males; however, the rate of suicide by firearms decreased significantly (r = −0.92, *P* = 0.001), and the rate of hanging increased significantly (r = 0.97, *P* < 0.001) in the United States. Between 2001 and 2010, poisoning was the most common suicide method among American females. During the same period in the United States, firearms were the second most common suicide method among American females, but the rate decreased significantly (r = −0.86, *P* = 0.001). In contrast, the rate of hanging increased significantly and steadily (r = 0.96, *P* < 0.001), but the rate of jumping decreased significantly (r = −0.90, *P* < 0.001) among American females (Figure 3).

**Figure 4:**
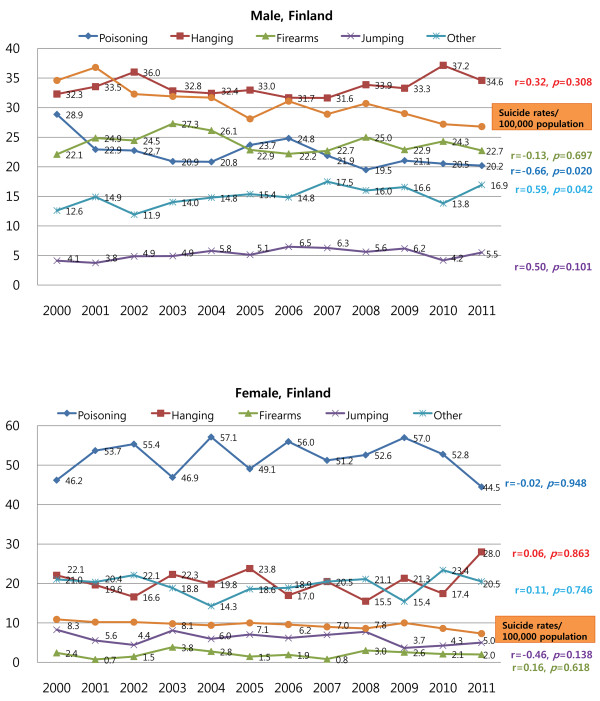
Trends in the suicide methods employed by Finnish males and females between 2000 and 2011.

Among both American males and females, there was a significant positive correlation between the suicide rate and the rate of hanging (r = 0.83, *P* = 0.002 and r = 0.96, *P* < 0.001, respectively), and a negative correlation between the suicide rate and the rate of suicide by firearms (r = −0.80, *P* = 0.003 and r = −0.84, *P* = 0.001, respectively). In addition, there was a significant negative correlation between the suicide rate and the rate of suicide by jumping in American females (r = −0.89, *P* < 0.001).In Finland, the most common method of suicide in males was hanging, while self-poisoning was the most common method of suicide in Finnish females during the study period. The trends for the changes in suicide method were not statistically significant for both males and females in Finland, and there was no significant correlation between suicide rate and the rate of any specific suicide methods (Figure 4).

## Discussion

We hypothesized that an increased use of a specific lethal suicide method was related to an increase in overall suicide rates. This hypothesis is supported by the findings of our present study, at least in case of South Korea. The proportion of highly lethal methods, namely hanging, increased, and the proportion of less lethal methods such as self-poisoning decreased among South Korean males and females, which were accompanied by a trend of increased annual suicide rates. This association between the proportion of suicides by hanging and annual changes in overall suicide rates was less prominent in the United States where the use of firearms, which is an even more lethal method, was replaced by hanging. This association was not observed in Finland, where annual changes in the suicide rate and the pattern of suicide methods were not prominent during the study period.

It should be noted that drug poisoning was a suicide method that has been commonly used in the past in South Korea, but has become less available in recent years due to strict regulations [12], and this may explain an increased use of hanging. In 2000, the Pharmacist Law was revised to restrict dispensing of prescription medications and in 2005, the Agrochemicals Control Act was revised to limit pesticide sales and purchases more strictly. These findings suggest that government restrictions limiting pesticide purchases and drug prescriptions are effective strategies for reducing suicide by these methods [13]. However, challenges remain for the prevention of suicide by hanging because of the difficulty of regulating and limiting the means to do so [14].

With regard to the rapid increase in suicide by hanging in South Korea since 2005, one consideration is the ability of celebrity suicides to induce copycat suicides, particularly in females [15]. The media’s reporting of suicide and its fictional portrayal on television are known to influence suicidal behavior, particularly the choice of method used [16-18]. Media guidelines for the sensational reporting of suicide are needed to reduce the portrayal of fictional suicides by lethal methods [19]. Suicide by hanging might be influenced by altered public perception of its acceptability [20]. Previous qualitative study of 22 suicide attempters that included 8 respondents who had attempted hanging showed that hanging was adopted for two main reasons, the anticipated nature of a death from hanging and accessibility [21]. Suicide attempters favoring hanging considered it as a clean, rapid and painless method with little awareness of dying and an easy method without the need of planning or technical knowledge. In contrary, respondents who rejected hanging recognized that it could be slow, painful, and messy method that needed technical knowledge for implementation. Authors suggested that prevention strategies should focus on countering perceptions of hanging as a clean, rapid, and painless method that is easily implemented [21]. Media could contribute to generate negative social perceptions of hanging [18]. Thus, suicide prevention agencies must work with the media to reduce the socio-cultural acceptability of hanging. In South Korea, which has the highest Internet penetration rate in the world [22], web-based programs to reduce the socio-cultural acceptability of hanging may be also effective. However, even if hanging suicides decreased by decreasing social acceptability of this lethal method, it could be replaced by another lethal method such as jumping and charcoal burning. Therefore, for an overall decrease in suicide rates, there is a need to adopt comprehensive, population-based approaches involving efforts to raise awareness about suicide prevention, reinforcement of social safety nets, promotion of socio-cultural beliefs that discourage suicide, and adequate prevention and treatment of depression and alcohol abuse, proven risk factors for suicide [23].

Firearms were the most common method of suicide among American males and the second most common method among American females. In the United States, several firearm policies have been enacted to limit access to firearms, especially for underage youth. In 1994, a federal law established 18 as the minimum legal age for the possession or purchase of handguns, including sales by gun owners who are not licensed dealers. American states vary in their requirements for gun ownership prohibition laws, such as banning of minors and people with history of felony, domestic violence offence, and mental or substance use problems [24]. Furthermore, gun safe storage laws, often referred to as child access prevention (CAP) laws, were enacted to prevent young people from gaining access to firearms. Most laws require gun owners to keep their guns in locked storage. A previous study demonstrating the effectiveness of CAP laws [25] found that the restriction of access to firearms is at least somewhat effective in decreasing the incidence of suicide by firearms and overall suicide rates in youth. Increased waiting period for the obtainment of handgun reduced suicide by firearm use among American adults over the age of 55 [26]. However, the reduction in suicide by firearms was offset by an increase in suicide by hanging, another lethal method, which resulted in no change in the overall suicide rate in the American population.

In Finland, the most common suicide methods were hanging in males and self-poisoning in females. In 1985, Finland established a research-based comprehensive national program for suicide prevention [10]. The suicide rate continued to increase until 1990, but then decreased by 20% between 1991 and 1996, and has been declining ever since. A comprehensive national program for suicide prevention in Finland included efforts to raise awareness about suicide prevention (e.g., recognizing the warning sign of suicide), efforts to reduce the social acceptability of suicide (e.g., effective and responsible regulations for reporting suicide), and efforts to limit the accessibility of a specific method of suicide (e.g., limiting access to firearms) [10]. Such comprehensive population-based approaches may have helped decrease suicide attempts by all means, rather than only decrease suicides attempts by specific methods, leading to an overall decrease in suicide rates. However, because the trends for changes in suicide method were not significant during the study period, we could not conclude with certainty that the decreased incidence of suicide attempts using lethal methods was related to the decrease in overall suicide rates in this country.

We must interpret the results of this study within the context of the following limitations. First, government case registries may underestimate the number of deaths due to suicide because many suicides may have been classified as “undetermined” deaths [27]. Second, although our focus was on the impact of suicide methods on annual changes in suicide rates, various other factors may have influenced the suicide rates, including social situations such as economic crises and celebrity suicides, which were not considered. Finally, subgroup analysis according to the ethnicity/race was not available for the heterogeneous United States population.

## Conclusions

Our present findings suggest that the increased use of hanging as a suicide method in South Korea is related to an increase in the overall suicide rate. Limiting access to lethal methods of suicide (e.g., firearms) is an effective strategy for reducing suicide by that particular method, but replacement by another lethal method (e.g., hanging) may lead to no overall decrease in the suicide rate. In addition, limiting access to relatively less lethal methods of suicide (e.g., drugs) can be easily replaced by more readily available and lethal methods of suicide (e.g., hanging). Therefore, the most fruitful approach to reducing overall suicide rates may be through population-based initiatives that reduce both the accessibility and the social acceptability of lethal methods of suicide.

## Competing interests

None of the authors have any financial interest in the study, or any other conflict of interest.

## Authors’ contributions

JPH designed the study, supervised the data collection, and assisted with writing the article. SP wrote the paper and carried out the statistical analysis. AL and MHA acquired the data and assisted with writing the article. All authors read and approved the final manuscript.
